# Variability in nutrient composition of the edible long‐horned grasshopper (*Ruspolia differens*) in Uganda and its potential in alleviating food insecurity

**DOI:** 10.1002/fsn3.3346

**Published:** 2023-04-11

**Authors:** Margaret Kababu, Collins K. Mweresa, Sevgan Subramanian, James P. Egonyu, Chrysantus M. Tanga

**Affiliations:** ^1^ International Centre of Insect Physiology and Ecology (icipe) Nairobi Kenya; ^2^ School of Agricultural and Food Sciences Jaramogi Oginga Odinga University of Science and Technology (JOOUST) Bondo Kenya

**Keywords:** edible grasshoppers, food security, functional food ingredient, geographical location, *Ruspolia differens*, vulnerable communities

## Abstract

*Ruspolia differens* Serville (Orthoptera: Tettigonidae) is a highly nutritious and luxurious insect delicacy that is consumed as a food source in many African countries. However, the nutrient profile of *R. differens* in different geographical regions have received limited research interest. Here, we provide comprehensive evidence of geographical impact on the nutrient profile of *R. differens* and its potential to meet the recommended dietary intake of the population. Our results demonstrated that proximate composition, fatty acids, amino acids, minerals, vitamins, and flavonoid contents of *R. differens* collected from five districts in Uganda varied considerably. The crude protein (28–45%), crude fat (41–54%), and energy (582–644 Kj/100 g) contents of *R. differens* exceed that reported from animal origins. The highest crude protein, crude fat, and carbohydrate contents of *R. differens* were recorded in Kabale, Masaka, and Kampala, respectively. A total of 37 fatty acids were identified with linoleic acid (omega‐6 fatty acid) being the most abundant polyunsaturated fatty acid in *R. differens* from Kabale, Masaka, and Mbarara. All essential amino acids were recorded in *R. differens*, particularly histidine with values exceeding the daily requirement for adults. Mineral and vitamin content differed significantly across the five districts. The highest quantity of flavonoids was recorded in *R. differens* from Hoima (484 mg/100 g). Our findings revealed that *R. differens* could be considered as functional food ingredients capable of supplying essential macro‐ and micronutrients that are critical in curbing the rising food insecurity and malnutrition in the regions.

## Graphical abstract

Long‐horned grasshoppers are consumed in over 21 African countries. Crude protein (28%–45%), crude fat (41%–54%), and energy (582–644 Kj/100 g) of grasshoppers are comparable to that of plant and animal origins. Grasshopper meal could be considered as functional food ingredients capable of supplying adequate essential macro‐ and micronutrients.

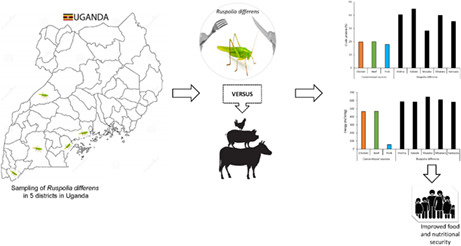

## 1 INTRODUCTION

*Ruspolia differens* Serville (Orthoptera: Tettigonidae) is an insect delicacy that is widely consumed as a main dish or snack in Eastern, Western, and Southern Africa (Agea et al., 2008; Mmari et al., 2017; van Huis, 2022). It has a rich profile of proteins (37%–54%) and essential amino acids that are not found in commonly consumed cereal proteins (Fombong et al., 2017; Ssepuuya et al., 2019; Zielińska et al., 2015). Its crude protein content exceeds values supplied by common animal protein sources (Orkusz, 2021). Thus, *R. differens* can serve as an alternative source of protein to supplement the increased demand for animal protein across the globe (Govorushko, 2019). *R. differens* contains fats (33%–49%) with high levels of polyunsaturated fatty acids (Fombong et al., 2017; Kinyuru et al., 2010; Nyangena et al., 2020). These polyunsaturated fatty acids are implicated in prophylaxis against autoimmune diseases, cancer, and osteoarthritis; at cellular level they influence the regulation of glucose levels, blood pressure, nervous system, blood clotting, and inflammatory reactions (Kapoor et al., 2021). The value of crude fats and energy in *R. differens* is higher than those obtained from pork, chicken, and beef (Orkusz, 2021). The high vitamins and mineral content of *R. differens* makes its consumption a critical option in addressing malnutrition and mineral deficiency especially in children and women of childbearing age in Sub‐Saharan Africa (Kinyuru et al., 2010; Mwangi et al., 2018; Ssepuuya et al., 2019). The grasshoppers contain flavonoids and phenols which are antioxidant compounds that provide anticancer, antiviral, antibacterial, and anti‐inflammatory activities in human (Cheseto et al., 2020; Ssepuuya et al., 2019). The quantities of these antioxidant compounds in *R. differens* are comparable to values recorded for different fruits and vegetables (Ssepuuya et al., 2019).

Like other edible insect species, *R. differens* is harvested from the wild during two annual swarming seasons (Mmari et al., 2017; van Huis, 2022). However, variations have been observed in the nutrient profile of the grasshoppers collected from different geographical locations. The proximate composition of *R. differens* differed among wild‐collected grasshoppers from Kenya, Uganda, and Zambia with a crude protein range of 38.9%–44.6%, crude fat (41.9%–49%), crude fiber (4.0%–12.7%), and carbohydrate (2.8%–8.4%) (Kinyuru et al., 2010; Siulapwa et al., 2012; Ssepuuya et al., 2017). Similarly, variations occurred in amino acid and fatty acid profile of *R. differens* collected from Uganda, Zambia, and Kenya (Fombong et al., 2017; Siulapwa et al., 2012; Ssepuuya et al., 2019). A similar trend was observed in the vitamin and mineral profile of *R. differens* collected from different countries (Fombong et al., 2017; Kinyuru et al., 2009; Ssepuuya et al., 2019). However, comparison of nutritional profile of wild‐collected *R. differens* from different regions in Uganda only recorded significant variations in mineral and vitamin B12 content of *R. differens* (Ssepuuya et al., 2019).

The variability of nutritional profile of insects is influenced by stage of development, origin, and diet (Govorushko, 2019; Kouřimská & Adámková, 2016). Nutritional composition of insects is influenced by their diets which implies that phytophagous *R. differens* feeding on host plants in different agro‐ecological zones would display considerable variation (Mwangi et al., 2018; Tang et al., 2019; van Huis, 2020). Nutritional composition of *R. differens* is known to be influenced by their diets (Lehtovaara et al., 2017; Rutaro, Malinga, Lehtovaara, Opoke, et al., 2018; Rutaro, Malinga, Lehtovaara, Valtonen, et al., 2018; Rutaro, Malinga, Opoke, Lehtovaara, et al., 2018). This study, therefore, evaluated the variability of nutritional composition of *R. differens* collected from different districts in Uganda where commercial harvesting and trade of *R. differens* is predominant. The study assessed the proximate composition, fatty and amino acids profile, minerals, vitamins, and flavonoid content of *R. differens* from different locations. Where data were available, these parameters were compared against common animal protein sources and daily requirements for human. The finding of this study will generate more evidence on the influence of geographical location of collection on nutritional composition of *R. differens* that provide information on the nutritional potential of *R. differens* to supplement the existing animal protein sources to curb global increase in food insecurity and malnutrition.

## 2 MATERIALS AND METHODS

### 2.1 Collection of *R. differens*

Raw *R. differens* were purchased from commercial harvesters from five different districts in Uganda: Kabale, Hoima, Mbarara, Kampala (Nakasero), and Masaka districts. These districts are known as the major areas where seasonal swamping of *R. differens* occurs within each year. The sample of the grasshoppers was purchased directly on‐site from the commercial harvesters from each of the five districts. The samples were processed by plucking the wings, legs, and ovipositors. The samples had a mixture of the major morphotypes (brown, green, pink, or brown infused with green colored morphs).

### 2.2 Sample preparation

*Ruspolia differens* samples were packed in polyethylene sterile Ziploc bags (SC Johnson brand, Size 15 × 13″) procured from a local supermarket in Kampala. The samples were labeled accordingly, packed with dry ice in cooler boxes, and sealed hermetically then transported by road to the laboratory in icipe Duduville campus in Nairobi (distance: 777.2 km; estimated time of travel: 16 h). Transportation of the grasshopper samples across the Kenya–Uganda border was facilitated by authorization permits obtained from Uganda's Ministry of Agriculture, Animal Industries and Fisheries Plant Quarantine and Inspection Services (License No: UQIS4471/93/PC (E)) and Kenya Plant Health Inspectorate Services (KEPHIS), Ministry of Agriculture, Fisheries and Cooperatives (Permit No: BIP/PS/22176/2021). *R. differens* samples were transferred into a freezer (Model: RZ41FARAEWW, Samsung, China) at *icipe* Duduville campus where they were stored at −20°C until further analysis.

A kilogram of frozen *R. differens* from each of the respective collection sites was allowed to thaw overnight under normal refrigeration at 5°C and rinsed with water to remove dirt. The samples were spread out evenly on aluminum foil and then oven dried (Model: SDO‐225‐CLAD‐F‐200 HYD; Wagtech Projects Ltd) at 60°C for 24 h (Fombong et al., 2017; Ochieng et al., 2022). The dried samples were ground using an electronic blender (Preethi TRIO, 500w, MG182/00), packed into Ziploc bags, and stored in a freezer for 24 h at −20°C prior to analysis at *icipe's* Behavioral and Chemical Ecology Unit (BCEU).

### 2.3 Data collection

#### 2.3.1 Determination of proximate composition of *R. differens*

Proximate composition was determined using the official methods of Association of Official Analytic Chemists (AOAC, 2012). Ash content was determined using a gravimetric method in a muffle furnace at 550°C for 3 h while moisture content was estimated as moisture loss after drying in an air oven at 105°C for 3 h. Kjeldahl method was used to determine the crude protein content of the samples following digestion in concentrated sulfuric acid. This was then computed using a 6.25 nitrogen‐to‐protein conversion factor. Soxhlet extraction method was used for the extraction of crude fat while crude fiber content was determined by acid digestion and evaluated by loss on ignition (Magara et al., 2019; Ochieng et al., 2022). The content of carbohydrates in each of the samples was estimated by subtracting the ash, moisture, fat, and protein content from 100%. Total energy (kJ/100 g) was computed using the formula: total energy = 4 × carbohydrate (%) + 4 × protein (%) + 9 × fat (%) (FAO, 2003).

#### 2.3.2 Determination of fatty acid content of *R. differens*

Fat extraction from the samples was conducted by adding 1 g of sample into 15‐mL falcon tube. The sample was mixed with 10 mL DCM: MeOH (2:1) in a hood. The mixture was vortexed for 10 s followed by sonication for 20 min and then allowed to stand for 1 h. The mixture was centrifuged for 10 min at 4800 *g*, then filtered into clean falcon tubes, and allowed to evaporate overnight in a hood until all solvents evaporated and crude oil extract remained.

Fatty acid methyl esters (FAMEs) were extracted as previously described by Cheseto et al. (2020). A quantity of 300 mg of oil extract was weighed into clean narrow neck vials. One and a half milliliters of sodium methoxide prepared by dissolving 2 g of sodium methoxide into 20 mL of dry methanol was added to the sample. The mixture was vortexed for 1 min, sonicated for 10 min, and then incubated in a water bath at 70°C for 1 h. Distilled deionized water (100 μL) was added to the mixture to quench the reaction and then vortexed for 1 min. GC‐grade hexane (1000 μL; Sigma‐Aldrich) was used to extract resultant FAMEs. A milliliter of hexane was added to the mixture, vortexed for 20 s, and then transferred to Eppendorf tubes prior to centrifugation at 16,000 *g* for 20 min. A quantity of 100 μL of supernatant was filtered into clean vials and dried through anhydrous sodium sulfate on insert fitted tips, followed by the addition of 900 μL hexane. The supernatant was analyzed using gas chromatography and mass spectrometry (GC–MS) on a 7890A gas chromatograph linked to a 5975C mass selective detector (Agilent Technologies Inc.). The GC was fitted with a (5%‐phenyl)‐methylpolysiloxane (HP5 MS) low bleed capillary column (30 m × 0.25 mm id, 0.25 μm film; J &W). The carrier gas was helium at a flow rate of 1.25 mL/min. Oven temperature was set to rise from 35°C to 285°C. The initial temperature was maintained for 5 min which rose at 10°C/min to 280°C at a hold time of 20.4 min. Quadrupole mass selective detector and ion source were maintained at temperatures of 230 and 180°C, respectively. Mass spectra of electron impact (EI) were obtained at an acceleration energy of 70 eV while fragment ions were analyzed between 40 and 550 m/z mass range in full scan mode. Filament delay period was fixed at 3.3 min. Serial dilution of authentic standard methyl octadecenoate (0.25–125 ng/ μL) was analyzed in full scan mode by GC–MS to produce a linear calibration curve (peak area against concentration) using the equation: [y = 5E + 07x + 2E + 07] which yielded *R* ^2^ = .9997. The generated regression equation was used to quantify the different fatty acids externally.

ChemStation B.02.02 acquisition software installed in an HP (Hewlett‐Packard: HP Z220 intel xeon) workstation was used to generate mass spectrum for each peak using the following integrators: initial threshold: 5, initial peak width: 0.1, initial area reject: 1, and shoulder detection: on. Identification of the compounds was done by comparison of mass spectral data and retention times with NIST 05, 08, and 11 authentic standards and reference spectra published by library‐MS databases. Analysis for each sample was done in triplicates.

#### 2.3.3 Determination of amino acid composition of *R. differens*

The amino acid composition of *R. differens* collected from the diverse localities was analyzed using a modified protocol previously described by Musundire et al. (2016). A quantity of 100 mg of each sample was weighed into a 5‐mL vial followed by 1.5 mL of 6 N HCL. The vial was capped following the introduction of nitrogen and then vortexed for 1 min. The samples were then placed in a GC oven at 110°C for 24 h to allow for complete hydrolysis. The samples were evaporated to dryness in a vacuum and then reconstituted in 1 mL 90:10 water: acetonitrile. The mixture was vortexed for 30 s, sonicated for 30 min, and then centrifuged at 14,000 rpm for 20 min. The supernatant was transferred into 1.5 mL vials and analyzed using UPLC‐MS/MS. An ACQUITY UPLC BEH C18 column (2.1 mm × 150 mm, 1.7 μm particle size; Waters Corp, Wexford, Ireland, oven temperature 45°C) was used to perform chromatographic separation of the samples. Cooling of the autosampler tray was done to a temperature of 5°C. Flow rate was maintained at 0.2 mL/min. The mobile phase consisted of water (A) and methanol (solvent B) which were both acidified with 0.01% formic acid. The utilized gradient system was: 0–2 min, 5% B; 2–4 min, 40% B; 4–7 min 40% B; 7–8.5 min, 60% B; 8.5–10 min, 60% B; 10–15 min, 80% B; 15–19 min, 95% B; and 24–26 min, 95% B. The UPLC was linked to a Waters Xevo TQ‐S electrospray ionization full scan MS in a positive ionization mode, m/z range of 40–2000 with a capillary voltage of 0.5 kV, sampling cone voltage of 30 V, source temperature of 150°C, desolvation temperature of 120°C, and nitrogen desolvation flow rate of 800 L/h. Massynx version 4.1 SCN 712 (Waters) was used for data acquisition. Amino acids were identified using mass spectrometric data, retention time, and coinjection of the hydrolysates with authentic standard amino acid mixture. External quantification of the amino acids present was done using amino acid standard solution (AAS 18) from Sigma‐Aldrich (Chemie GmbH). Each of the samples was analyzed in triplicate.

#### 2.3.4 Mineral analysis of *R. differens* samples

Mineral composition was determined using ICP‐OES (Inductively Coupled Plasma‐Optical Emission Spectrometer) analysis (Campbell & Plank, 1991; Horwitz, 2000). A quantity of 0.5 g of ground and thoroughly homogenized grasshopper samples were mixed with 8.0 mL of concentrated nitric acid and 2 mL 30% hydrogen peroxide in a digestion tube and then left to stand overnight in a fume hood. The digestion tube was placed into a block digester (Model: The BD50/BD28 Series Bock Digestion System, Seal Analytical limited) for serial digestion at 75, 120, 180, and 200°C at an interval of 30, 20, 20, and 10 min, respectively, until the solution was clear with no debris. The digestion tubes were removed from the block digester and allowed to cool. The digest was quantitatively transferred to 25‐ and 50‐mL falcon tubes, then diluted to the mark with 2% Nitric acid, and then taken to the ICP‐OES equipment (Model: Optima 2100DV, PerkinElmer) for mineral quantification. The operating conditions for ICP‐OES equipment were as follows: Radio frequency power (W), 1450; Plasma gas flow rate (L/min), 15; Auxiliary gas flow rate (L/min), 0.2; Nebulizer gas flow rate (L/min), 0.8; Sample flow rate (L/min), 1.5; View mode, axial; Read, peak area; Source equilibrium time (s), 10; Read delay (s), 10; replicates, 1; Background correction, 2‐point (manual point correction); Spray chamber, Scott type spray chamber; Nebulizer cross, Flow GemTip nebulizer (HF resistant); Detector CCD, CCD; Purge gas, nitrogen; Shear gas, air and Plasma gas, argon. The minerals were measured at the following wavelength (nm): Zn‐ 213.857, Mg‐285.213, Fe‐259.939, Mn‐257.61, P‐213.617, Mo‐202.031, K‐766.49, Al‐396.153, Cu‐224.7, Co‐ 228.616, and Ca‐317.933. ICP‐OES mix standard CatNo. 43843 (Sigma Aldrich) was used for quantification. External standard calibration method was applied, and serial dilution of the standards was performed using 2% nitric acid to obtain calibration standards of 400, 800, 2000, and 4000 μg/L. Calibration was done using Perkin Elmer Winlab 32 software. This analysis was done in triplicate.

#### 2.3.5 Determination of vitamin content of *R. differens*

The composition of water‐soluble vitamins was determined by Liquid Chromatography–Diode array detector (Thermo Fisher Scientific, 2010). Ground sample (100 mg) was weighed in a 50‐mL falcon tube and mixed with 25 mL of distilled water. The mixture was ultra‐sonicated for 15 min and then filtered into UPLC (Ultra Performance Liquid Chromatography) vials through 0.2 μm filters. The vials were capped and loaded into the UPLC autosampler for analysis. Stock solutions of 1.0 mg/mL were prepared by dissolving individual water‐soluble vitamins in distilled water with the exception of Vit B2 and Vit B9 which were dissolved in 5 mM potassium hydroxide and 20 mM potassium hydrogen carbonate, respectively. Four calibration standards were prepared from the mix at a concentration of 2, 5, 10, and 15 μg/mL. Chromatography was performed using Nexera Liquid Chromatograph LC‐30 AC with Nexera column oven CTO‐30A with the following specifications: Detector, Diode Array Detector; Column, Phenomenex Synergi 2.6 μm plar C18‐100 mm × 3.0 mm; Column Oven temperature, 30°C; LC program of 12 min Run time, Mobile Phase A: 25 mM Phosphate buffer, Mobile Phase B: 7:3 v/v Acetonitrile‐Mobile phase A; Flow Rate of 0.4 mL/min and distilled water as Column Flushing Solution. Extraction, detection, identification, and quantification of each sample were done in triplicate.

The composition of fat‐soluble vitamins (retinol and tocopherols) was determined by HPLC (High‐Performance Liquid Chromatography) (Bhatnagar‐Panwar et al., 2013; Hosotani & Kitagawa, 2003). A quantity of 0.5‐g sample of ground grasshopper was weighed in a 25‐mL tube in triplicate, mixed with 6 mL ethanol with 0.1% BHT (Butylated Hydroxytoluene), and homogenized for a minute. A quantity of 120 μL of potassium hydroxide 80% (w/v) was added to the solution and mixed by vortexing. The mixture was incubated for 5 min at 85°C, removed from the water bath, and immediately cooled in ice. Four milliliters of deionized water was added to each tube and then mixed in a vortex. Five milliliters of hexane was added to the tubes and then mixed in a vortex. The sample was centrifuged for 5 min at 3429 *g*. The upper phase (hexane) was transferred to a centrifuge tube using Pasteur pipette. This was extracted three more times with 4 × 3 × 3 mL hexane and the extract pooled into a 25‐mL tube. Additional 5‐mL deionized water was added to the extract, vortexed for a minute, and centrifuged at 3429 *g* for 5 min. The hexane layer was recovered into clean test tubes and then evaporated to complete dryness under nitrogen in the N‐Evap. This was then reconstituted 1 mL of methanol: tetrahydrofuran (85:15 v/v), vortexed and sonicated for 30 seconds, and then transferred to 0.8 mL HPLC vials. HPLC system used was Shimadzu Nexera UPLC system linked to SPD‐M2A detector with Reverse phase gradient HPLC method with the following specifications: Oven temperature, off; YMC C30, carotenoid column (3 μm, 150 × 3.0 mm, YMC); injection volume, 10 μL; Mobile phase A: Methanol/tert‐butyl methyl ether/water (85:12:3, v/v/v, with 1.5% ammonium acetate in the water); Mobile phase B: methanol/tert‐butyl methyl ether/water (8:90:2, v/v/v, with 1% ammonium acetate in the water); and a total flow rate of 0.4 mL/min. Extraction, detection, identification, and quantification of each sample were done in triplicate.

#### 2.3.6 Determination of flavonoids

Flavonoid content of *R. differens* samples was determined using Aluminum Chloride calorimetry (Dewanto et al., 2002; Singleton & Rossi, 1965; Zhishen et al., 1999). Sample extraction was done by weighing 0.5 g into a clean propylene tube. This was followed by the addition of 10 mL of 80% methanol and then shaking in a mechanical shaker at 25°C for 24 h. The mixture was centrifuged for 10 min at 4571 *g* and the supernatant aliquot was taken out for the determination of total flavonoids. Approximately 20‐μL aliquot of sample extract or standard solution of catechin (10, 20, 40, 60, 80, and 100 μg/mL) was pipetted into microtiter well and then mixed with 80 μL of deionized distilled water. Ten microliters of 5% NaNO_2_ was added to the mixture and mixed by priming. After 5 min, 10 μL of 10% ALCL_3_ was added and mixed gently by priming. Five minutes later, 80 μL of 2 M NaOH was added to the mixture and mixed gently by priming. The reaction was incubated at room temperature for 30 min. The absorbance of the samples and standards were read against the reagent blank using a Ultraviolet–visible spectrophotometer at a wavelength setting of 510 nm. Standard calibration (0.01–0.02–0.04–0.06–0.08–0.1 mg/mL) curve of catechin was plotted in 80% methanol. Extraction, detection, identification, and quantification of each sample were done in triplicate.

### 2.4 Data analysis

All analyses were performed using R version 4.1.2 (R Core Team, 2020), statistical software. Shapiro–Wilk's and Bartlett's tests were used to test for normality and homogeneity of data, respectively. All normal and homogenous data were subjected to one‐way analysis of variance (one‐way ANOVA) to test for differences in nutritional composition of *R. differens* collected from different sites. Nonnormal data were subjected to log transformation prior to ANOVA. Mean separation was done using Tukey's Honest Significant Difference (HSD), post hoc test where the differences were significant. Normal nonhomogeneous data were subjected to Welch ANOVA and differences in means separated using Games–Howell test where there were significant variations. All effects were considered significant at *p* < .05.

## 3 RESULTS

### 3.1 Proximate composition of *R. differens*

Proximate composition of *R. differens* varied across the geographical locations (Table 1). The highest crude protein from *R. differens* was obtained from Kabale, while higher values of crude fiber and carbohydrates were observed in Mbarara and Kampala, respectively. *Ruspolia differens* obtained from Masaka recorded the highest energy (645 ± 2.2 kj/100 g) and moisture (5.3 ± 0.0%) content while those from Hoima recorded the highest ash (2.8 ± 0%) content. The crude protein, crude fat, and energy content of *R. differens* collected from the various sites exceeded the values recorded for common animal protein sources (Orkusz, 2021; Williams et al., 2016).

**TABLE 1:** Proximate composition (% DM basis) of *Ruspolia differens* (mean ± SE) collected from different geographical locations versus common animal protein sources.

Component	Proximate composition (% DM)								*F* value	Df	*p* Value
	Common protein sources (Orkusz, 2021; Williams et al., 2016)			*R. differens* collected from different locations							
	Chicken	Beef	Pork	Hoima	Kabale	Masaka	Mbarara	Kampala			
Crude protein	19.8 ± 1.4	20.0 ± 1.5	17.8 ± 1.7	40.4 ± 0.4 a	44.8 ± 0.3 b	28.2 ± 1.7 c	40 ± 0.3 a	35.3 ± 0.3 d	63.6	4, 10	<.001
Crude fat	4.8 ± 1.7	9.8 ± 3.9	8.8 ± 4.4	42.6 ± 0.8 a	41 ± 0.6 a	54.3 ± 0.4 b	46.7 ± 0.4 c	41.8 ± 0.7 a	87.1	4, 10	<.001
Crude fiber				5.0 ± 0.2 ab	4.7 ± 0.2 ab	4.6 ± 0.2 a	5.3 ± 0.1 b	4.5 ± 0.1 a	5.4	4, 10	.014
Carbohydrates				10.7 ± 1.2 a	8.6 ± 0.8 a	10.8 ± 1.4 a	7.5 ± 0.2 a	16.2 ± 1.1 b	10.9	4, 10	.001
Ash	1.2	0.9	0.8	2.8 ± 0 a	2.7 ± 0.0 a	1.4 ± 0.1 c	2.3 ± 0.0 ab	1.9 ± 0.1 bc	121.2	4, 10	<.001
Moisture				3.5 ± 0.1 a	3.0 ± 0.1 b	5.3 ± 0.0 c	3.6 ± 0.1 a	4.7 ± 0.1 d	154.6	4, 10	<.001
Energy (Kj/100 g)	466.5	468.6	54.4	588 ± 3.6 a	583 ± 2.5 a	644 ± 2.2 b	610 ± 2.0 c	582 ± 3.3 a	90.8	4, 10	<.001

*Note*: Mean (± SE) in the same row followed by similar letter (s) is not significantly different (Tukey's HSD test: *p* < .05).

### 3.2 Fatty acid composition of *R. differens*

A total of 37 fatty acids were identified from oils extracted from *R. differens* samples analyzed out of which 51%, 38%, and 11% were saturated (SFA), monounsaturated (MUFA), and polyunsaturated fatty acids (PUFA), respectively (Table 2). The quantities of fatty acids varied significantly across the geographical areas of collection.

**TABLE 2:** Mean (± SE) of fatty acid composition (μg/g of oils) of *Ruspolia differens* collected from different geographical locations.

Fatty acid methyl ester	Common name	Common protein sources (%) (Kasprzyk et al., 2015; Muchenje et al., 2009; Riovanto et al., 2012)			Quantity of fatty acid methyl esters (μg/g) in *R. differens* collected from diverse location					*F* value	Df	*p* Value
		Chicken	Beef	Pork	Hoima	Kabale	Mbarara	Masaka	Kampala			
Saturated fatty acids (SFA)												
Methyl docosanoate	Behenic acid				402.49 ± 75.3 a	0.31 ± 0.0 b	2.42 ± 0.1 c	77.29 ± 32.8 d	0.14 ± 0.0 e	945	4, 10	<.001
Methyl eicosanoate	Arachidic acid				32.00 ± 0.8 a	0.06 ± 0.0 b	27.77 ± 7.3 a	38.60 ± 6.7 a		324.9	3, 8	<.001
Methyl heneicosanoate					797.03 ± 55.6 a	7.86 ± 0.6 b	9.32 ± 0.8 b	1.37 ± 0.0 c	0.96 ± 0.1 c	831.1	4, 10	<.001
3‐methyl‐heptacosanoiate						6.71 ± 0.2 a			122.54 ± 3.5 b	4917	1, 4	<.001
Heptadecanoic acid, 3‐methyl‐, methyl ester					514.89 ± 39.8 a	42.19 ± 3.2 a				393.9	1, 4	<.001
Methyl 16‐pentacosenoate						2558.34 ± 27.5 a	1.94 ± 0.1 b			43266	1, 4	<.001
Methyl 18‐methylnonadecanoate					3.16 ± 0.1 a	8.16 ± 0.4 a			5.89 ± 0.1 c	188	2, 6	<.001
Methyl 24‐methyl‐hexacosanoate						4.99 ± 1.0 a	1.13 ± 0.1 b		0.19 ± 0.0 c	147	2, 6	<.001
Methyl dodecanoate	Lauric acid			0.18 ± 0.0	5.09 ± 0.2 a	1892.24 ± 104.4 b	1141.53 ± 58.9 c	1.72 ± 0.1 d	0.57 ± 0.0 e	4430	4, 10	<.001
Methyl hexadecanoate	Palmitic acid	22.49 ± 1.3	22.3 ± 0.7	23.86 ± 0.4	4.05 ± 0.3 a	18.50 ± 0.7 b	918.21 ± 91.6 c	1490.29 ± 68.4 d	1053.22 ± 26.6 c	2024	4, 10	<.001
Methyl octadecanoate	Stearic acid	7.9 ± 1.0	18.75 ± 0.6	11.96 ± 0.5			15.29 ± 0.8 a	8.54 ± 0.5 b		65.3	1, 4	.001
Methyl tetradecanoate	Myristic acid	0.88 ± 0.2	1.51 ± 0.2	0.14 ± 0.1	7.93 ± 0.6 a	9.58 ± 1.4 a	7.68 ± 0.4 a	81.92 ± 4.4 b	3.78 ± 0.4 c	152.7	4, 10	<.001
Methyl nonadecanoate					3.68 ± 0.8 a	5.94 ± 4.4 a	1323.27 ± 13.9 b	8.46 ± 0.4 a	6.85 ± 0.4 a	51	4, 10	<.001
Methyl octanoate						74.20 ± 8.8 a	5.73 ± 0.3 b	0.15 ± 0.0 c		1063	2, 6	<.001
14‐methyl‐pentadecanoate					3.54 ± 0.8		5.10 ± 0.1			3.4	1, 4	.14
Methyl pentadecanoate					6.50 ± 0.2 a		10.24 ± 1.4 a	2.94 ± 0.4 b	27.01 ± 0.5 c	86.3	3, 8	<.001
Methyl tetracosanoate					4.46 ± 0.8 a	16.97 ± 1.1	1302.26 ± 37.7 c	4.29 ± 0.2 a	11.31 ± 1.8 b	417.4	4, 10	<.001
Methyl tricosanoate	Tricosylic acid				3.09 ± 0.1 a	2.07 ± 0.1 b	4.18 ± 0.2 c	1.55 ± 0.1 d	2.74 ± 0.1 a	98.8	4, 10	<.001
Methyl tridecanoate	Tridecylic acid					1.19 ± 0.0 a	2.96 ± 0.1 c	2.28 ± 0.1 c	1.86 ± 0.1 d	92.2	3, 8	<.001
∑SFA					1787.91 ± 175.4	4649.31 ± 153.8	4779 ± 213.8	1719.11 ± 114.1	1237.06 ± 33.6			
Monounsaturated fatty acids (MUFA)												
Methyl hexadec‐9‐enoate	Palmitoleic acid	2.34 ± 0.5	2.26 ± 0.1	2.51 ± 0.4		61.50 ± 1.8 a	2.21 ± 0.1 b	175.39 ± 26.8 c	14.57 ± 0.7 d	535.2	3, 8	<.001
Methyl 9Z‐hexadecenoate	elaidolinolenic acid				36.61 ± 2.7 a		1.99 ± 0.1 b			1033	1, 4	<.001
Methyl cis‐13‐eicosenoate	Paullinic acid				9.84 ± 1.7 a		276.93 ± 95.0 b		0.63 ± 0.0 c	198.9	2, 6	<.001
Methyl 11‐octadecenoate	vaccenic acid					0.03 ± 0.0 a	0.04 ± 0.0 a	5.01 ± 0.2 b		1843	2, 6	<.001
Methyl 13Z‐docosenoate	Erucic acid					26.10 ± 2.6 a	4.25 ± 1.2 b	3.89 ± 0.1 b	0.34 ± 0.0 c	158.4	3, 8	<.001
Methyl 6Z‐octadecenoate	Petroselinic acid				0.18 ± 0.0 a		0.38 ± 0.0 b	6.39 ± 0.2 c		1063	2, 6	<.001
Methyl 7‐octadecenoate						566.35 ± 22.3						
Methyl 9Z‐octadecenoate	Oleic acid	31.2 ± 2.9	31.07 ± 1.0	47.37 ± 0.9	12.08 ± 0.6 a	35.67 ± 3.7 b	37.35 ± 1.8 b	1231.75 ± 47.7 c	2.56 ± 0.1 d	1388	4, 10	<.001
Methyl cis‐10‐heptadecenoate					1.85 ± 0.1 a	3.86 ± 0.1 b	0.55 ± 0.0 c	10.37 ± 0.5 d	38.49 ± 1.5 e	1909	4, 10	<.001
Methylcis‐10‐Nonadecenoate					0.86 ± 0.1 a	203.80 ± 8.7 b	223.17 ± 10.5 b	9.55 ± 0.4 c	0.75 ± 0.1 a	2115	4, 10	<.001
Methyl cis‐11‐eicosenoate					15.26 ± 1.3 a	0.12 ± 0.0 b	0.11 ± 0.0 b	22.06 ± 3.2 a		409.4	3, 8	<.001
Methyl cis‐13‐octadecenoate						1.58 ± 0.1 a		13.61 ± 0.3 b	2.20 ± 0.1 c	717.5	2, 6	<.001
Methyl cis‐9‐tetradecenoate	Myristoleic acid					233.23 ± 5.8 a	115.02 ± 4.1 b		2.49 ± 0.0 c	8473	2, 6	<.001
Methyl 11Z‐tetradecenoate						1.96 ± 0.2 a		0.41 ± 0.1 b		55.0	1, 4	.002
∑MUFA					76.68 ± 6.5	1134.2 ± 45.3	662 ± 112.8	1478.4 ± 79.5	62.03 ± 2.4			
Polyunsaturated fatty acids (PUFA)												
3,7,11,15‐tetramethyl‐6,10,14‐Hexadecatrienoate [R‐(E,E)]‐	Phytanic acid methyl ester				2.41 ± 0.2 a	9.15 ± 0.6 b				172.1	1, 4	<.001
Methyl (6Z,9Z,11E)‐octadecatrienoate									1518.85 ± 177.6			
Methyl (9Z,11E,13E)‐octadecatrienoate	α eleostearic acid					12.71 ± 1.2 a	3.39 ± 0.1 b	4.58 ± 0.1 c	3.68 ± 0.3 bc	92.1	3, 8	<.001
Methyl (9Z,12Z)‐octadecadienoate	Linoleic acid	24.2 ± 3.5	2.21 ± 0.2	8.72 ± 0.5		2228.11 ± 107.5 a	593.89 ± 40.7 b	2322.43 ± 60.4 a	7.34 ± 0.2 c	2056	3, 8	<.001
∑PUFA					2.41 ± 0.2	2249.97 ± 109.3	597.28 ± 40.8	2327.01 ± 60.5	1529.87 ± 178.1			

*Note*: Mean (± SE) in the same row followed by similar letter (s) is not significantly different (Tukey's HSD test: *p* < 0.05).

The most abundant SFA was methyl 16‐pentacosenoate (2558.34 ± 27.5 μg/g) which was detected in *R. differens* collected from Kabale (*F*_1,4_ = 43266, *p* < .001). Palmitic acid (methyl hexadecanoate) was the second most abundant SFA with quantities that differed significantly among *R. differens* collected from the different sites (*F*_4,10_ = 2024, *p* < .001); higher quantities were recorded in *R. differens* collected from Masaka (1490.29 ± 68.4 μg/g) and Kampala (1053.22 ± 26.6 μg/g).

Oleic acid (methyl 9Z‐octadecenoate) was the most prevalent MUFA. A significantly higher amount of oleic acid was detected in *R. differens* collected from Masaka (1231.75 ± 47.7 μg/g) compared to the other locations (*F*_4,10_ = 1388, *p* < .001). Linoleic acid was the most abundant PUFA with significantly high amounts (*F*_3,8_ = 2056, *p* < .001) extracted from *R. differens* collected from Masaka (2322.43 ± 60.4 μg/g), Kabale (2228.11 ± 107.5 μg/g), and Mbarara (593.89 ± 40.7 μg/g).

### 3.3 Amino acid composition of *R. differens*

Nine essential and eight nonessential amino acids were detected in *R. differens* samples analyzed (Table 3). The quantities of methionine (*F*_4,10_ = 4.38, *p* = .026) and lysine (*F*_4,10_ = 4.19, *p* = .030) varied by area of collection while the quantities of the other essential amino acids did not differ. Quantities of proline (*F*_4,10_ = 3.52, *p* = .048) and glycine (*F*_4,10_ = 3.66, *p* = .043) varied by area of collection while the quantities of other nonessential amino acids were not different. The leucine content of a gram of *R. differens* analyzed was higher than quantities obtained in common animal protein sources and exceeded the daily requirements for human adults (Orkusz, 2021; Rumpold & Schlüter, 2013a).

**TABLE 3:** Amino acid content of *Ruspolia differens* (Mean ± SE) collected from diverse geographical sites versus common protein sources and daily requirements for human adults.

Amino acid	Daily requirement for human adults (mg/day) (Rumpold & Schlüter, 2013a; WHO, 2007)	Quantity of amino acids (mg/g)								*F* value	Df	*p* Value
		Common protein sources (Orkusz, 2021)			Quantity of amino acids (mg/g) of *R. differens* collected from different locations							
		Chicken	Beef	Pork	Hoima	Kabale	Mbarara	Masaka	Kampala			
Essential amino acids												
Leucine	59.0	14.1	16.8	14.3	99.43 ± 20.0	93.18 ± 25.7	52.83 ± 21.6	74.7 8 ± 23.2	92.39 ± 38.0	0.51	4, 10	.729
Arginine		7.4	13.1	10.9	4.28 ± 0.6	5.60 ± 0.1	2.87 ± 1.2	4.18 ± 0.7	5.28 ± 0.1	3.07	4, 10	.068
Lysine	45.0	18.1	18.4	14.8	4.59 ± 2.3 a	10.36 ± 0.3 b	4.02 ± 2.1 a	5.71 ± 1.5 ab	10.54 ± 0.1 b	4.19	4, 10	.030
Threonine	23.0	16.3	9.5	9.7	2.18 ± 0.3	4.21 ± 3.0	1.37 ± 0.4	1.49 ± 0.3	1.21 ± 0.0	0.84	4, 10	.532
Valine	39.0	12	10.4	9.2	4.08 ± 0.2	5.19 ± 0.3	2.97 ± 1.3	3.13 ± 0.4	5.54 ± 0.4	3.38	4, 10	.053
Histidine	15.0	8.4	7.1	5.8	2.55 ± 0.2	3.37 ± 0.1	1.70 ± 0.7	2.70 ± 0.5	3.25 ± 0.1	3.06	4, 10	.068
Isoleucine	30.0	11.5	9.8	8.2	9.94 ± 2.0	9.31 ± 2.6	5.28 ± 2.2	7.47 ± 2.4	9.23 ± 3.8	0.51	4, 10	.729
Methionine	16.0	5.6	5.6	4.9	1.49 ± 0.1 ab	2.34 ± 0.2a b	1.15 ± 0.7 ab	1.04 ± 0.2 a	2.72 ± 0.3 b	4.38	4, 10	.026
Phenylalanine		6.9	9.1	7	3.48 ± 0.7	5.17 ± 0.6	3.29 ± 2.2	2.82 ± 1.1	8.68 ± 1.4	3.18	4, 10	.063
Nonessential amino acids												
Alanine		12.9	12.1	10.6	1.68 ± 0.6	0.81 ± 0.1	2.24 ± 1.2	1.73 ± 1.2	0.63 ± 0.1	0.63	4, 10	.655
Aspartic acid		19.3	18.6	15.4	5.16 ± 0.6	3.19 ± 0.2	3.58 ± 0.9	3.50 ± 0.7	3.10 ± 0.1	0.17	4, 10	.171
Cysteine	6.0	2.5	2.7	2.1	0.36 ± 0.2	0.34 ± 0.1	0.28 ± 0.1	0.42 ± 0.1	0.34 ± 0.0	0.34	4, 10	.842
Glutamic acid		31.3	31.7	25.4	1.15 ± 0.2	1.83 ± 0.2	2.86 ± 1.1	1.63 ± 0.5	1.63 ± 0.1	1.32	4, 10	.327
Proline		9.2	7.8	6.6	4.16 ± 0.2 abc	5.65 ± 0.4 a	3.25 ± 1.0 c	3.81 ± 0.5 bc	5.43 ± 0.2 ab	3.52	4, 10	.048
Glycine		11.9	10.1	9.2	3.36 ± 0.2 a	2.12 ± 0.1 bc	2.21 ± 0.7 bc	3.12 ± 0.0 ab	2.09 ± 0.1 c	3.66	4, 10	.043
Tyrosine		6.5	7.5	6.2	3.38 ± 0.3	4.75 ± 1.0	2.50 ± 1.5	2.22 ± 0.5	5.85 ± 0.7	2.94	4, 10	.076
Serine		7.8	8.4	6.2	2.57 ± 0.2	1.58 ± 0.1	1.79 ± 0.6	2.28 ± 0.1	1.59 ± 0.1	2.66	4, 10	.095

*Note*: Mean (± SE) in the same row followed by similar letter (s) is not significantly different (Tukey's HSD test: *p* < .05).

### 3.4 Mineral composition of *R. differens* collected from different geographical locations

Several macro‐ and microminerals were obtained in varying quantities from *R. differens* collected from the five districts (Table 4). Potassium was the most abundant macromineral (198–871 mg/100 g) with significantly higher quantities obtained in *R. differens* collected from Kabale compared to the other locations (*F*_4,10_ = 177.9, *p* < .001). Iron was the most prevalent micromineral (7.2–155 mg/100 g), significantly higher quantities were recorded in *R. differens* collected Hoima compared to other locations (*F*_4,4.06_ = 2435.5, *p* < .001). The quantities of zinc and iron in *R. differens* analyzed exceed the quantities obtained from beef, pork, and chicken and the recommended daily intake for humans (FAO, 2004; Orkusz, 2021; Rumpold & Schlüter, 2013a).

**TABLE 4:** Mineral content of *Ruspolia differens* (Mean ± SE) collected from different geographical locations, common protein sources, and recommended daily intake.

Mineral	Recommended daily intake (mg/day) (FAO, 2004; Rumpold & Schlüter, 2013a)	Mineral content (mg/100 g)								*F* value	Df	*p* Value
		Common protein sources (Orkusz, 2021)			*R. differens* from different locations							
		Chicken	Beef	Pork	Hoima	Kabale	Masaka	Mbarara	Kampala			
Phosphorus	700	227.5	212.0	130.4	96.1 ± 4.7 a	99.4 ± 5.7 a	38.8 ± 0.4 b	99.2 ± 0.4 a	510 ± 18.4 c	2344.4	4, 4.57^†^	<.001
Potassium	2000	359.5	382	293.6	526 ± 12.9 a	871 ± 30.4 b	198 ± 6.6 c	641 ± 14.8 d	466 ± 18.8 a	177.9	4, 10	<.001
Magnesium	220	29.5	26.0	15.6	54.6 ± 1.5 a	46.8 ± 0.8 ac	16.1 ± 1.1 b	47.1 ± 1.8 ac	44.5 ± 2.6 c	76.0	4, 10	<.001
Calcium	1300	6.5	4.0	4.1	50.1 ± 1.7 a	39.1 ± 0.4 b	19.3 ± 0.8 c	58.9 ± 2.6 d	33.2 ± 0.5 b	107	4, 10	<.001
Sodium	500	73	52	50	65.7 ± 1.7 a	129 ± 6.6 b	27.4 ± 1.7 c	98.1 ± 5.1 d	56.7 ± 2.3 a	93.8	4, 10	<.001
Aluminum					22.5 ± 1.1 a	15.9 ± 0.2 b	4.37 ± 0.2 c	17.4 ± 0.4 b	15.7 ± 0.8 b	97.6	4, 10	<.001
Zinc	7.2	1.0	2.9	2.2	16.8 ± 0.5 a	11.1 ± 0.2 b	6.3 ± 1.9 c	12.3 ± 0.6 b	17.2 ± 0.5 a	22.3	4, 10	<.001
Iron	27.4	0.6	3.1	0.9	155 ± 6.7 a	80.1 ± 1.7 b	7.2 ± 0.2 c	20.9 ± 1.1 d	30.5 ± 0.0 e	2435.5	4, 4.06^†^	<.001
Copper	1.5	0.1	0.1	0.1	2.8 ± 0.2 a	2.7 ± 0.0 a	1.67 ± 0.1 b	2.27 ± 0.1 c	2.17 ± 0.1 c	22.7	4, 10	<.001
Manganese	2.3	0.0	0.0	0.0	4.1 ± 0.1 ab	3.6 ± 0.1 ac	1.07 ± 0.1 d	4.67 ± 0.2 b	3.37 ± 0.1 c	118.3	4, 10	<.001
Molybdenum					0.2 ± 0.0 ab	0.25 ± 0.0 a	0.2 ± 0.0 b	0.26 ± 0.0 a	0.14 ± 0.01 c	23.2	4, 10	<.001

*Note*: Mean (± SE) in the same row followed by similar letter (s) is not significantly different (Tukey's HSD test/^†^Games–Howell test: *p* < .05).

### 3.5 Vitamin content of *R. differens* collected from different geographical locations

Vitamin content of *R. differens* differed significantly by area of origin (Table 5). The most abundant vitamins obtained were pantothenic acid (165.2–248 mg/100 g), folic acid (79–137.6 mg/100 g), and pyridoxine (87.9–151.5 mg/100 g). Significantly higher quantities of pantothenic acid were obtained in *R. differens* from Hoima (*F*_4,10_ = 25.2, *p* < .001) while significantly higher amounts of folic acid (*F*_4,4.69_ = 285.6, *p* < .001) and pyridoxine (*F*_4,10_ = 148.5, *p* < .001) occurred in *R. differens* collected from Kabale. The quantities of niacin, pantothenic acid, pyridoxine, folic acid, and vit B12 obtained from *R. differens* samples exceeded the values contained in pork, chicken, and beef; and the daily requirements in adults (FAO, 2004; Orkusz, 2021).

**TABLE 5:** Vitamin content of *Ruspolia differens* (Mean ± SE) collected from different geographical locations, common protein sources, and recommended daily intake.

Vitamin	Daily requirement in adults (mg/day) (FAO, 2004)	Vitamin content (mg/100 g)								*F* value	Df	*p* Value
		Common protein sources (Orkusz, 2021)			*R. differens* from different locations							
		Chicken	Beef	Pork	Hoima	Kabale	Masaka	Mbarara	Kampala			
Vit A/Retinol	0.6	13 μg/100 g	11 μg/100 g	0.0	0.8 ± 0.0 a	0.28 ± 0.1 b	0.7 ± 0.0 a	1.0 ± 0.3 c	0.3 ± 0.1 b	124.6	4, 10	<.001
Vit B2/Riboflavin	1.3	0.2	0.3	0.2	2.6 ± 0.2 a	1.3 ± 0.1 b	1.2 ± 0.1 b	6.3 ± 0.2 c	1.1 ± 0.0 b	275.8	4, 10	<.001
Vit B3/niacin	16	7.8	5.5	4.9	26.4 ± 1.33 a	29.2 ± 1.06 a	—	21.0 ± 0.47 b	—	16.7	2, 6	.004
Vit B5/Pantothenic acid	5				239.9 ± 6.4 a	234.5 ± 12 a	174.5 ± 5.6 b	165.2 ± 1.9 b	248.1 ± 9.4 a	25.2	4, 10	<.001
Vit B6 /Pyridoxine	1.7	0.4	0.3	0.3	101.7 ± 2.4 a	151.5 ± 2.8 b	93.4 ± 2.1 ac	87.9 ± 1.2 c	91.0 ± 2.0 c	148.5	4, 10	<.001
Vit B9/folic acid	0.4				135.2 ± 2.4 a	137.6 ± 7.1 a	79.0 ± 0.8 b	134.3 ± 1.4 a	107.8 ± 1.4 c	285.6	4, 4.69^†^	<.001
Vit B12/cobalamin	2.4 μg/day	0.4 μg/100 g	1.4 μg/100 g	0.6 μg/100 g	3.2 ± 0.2 a	3.07 ± 0.1 a	2.19 ± 0.1 b	3.3 ± 0.2 a	2.0 ± 0.1 b	12.7	4, 10	.001
Gamma tocopherol	10 μg/100 g	0.3	0.2	0.3	1.1 ± 0.0 a	1.1 ± 0.0 a	—	—	1.1 ± 0.01 a	1.5	2, 6	.288
α‐tocopherol					0.5 ± 0.0 a	01 ± 0.0 b	0.1 ± 0.0 b	0.1 ± 0.0 b	0.5 ± 0.01 a	284	4, 4.9^†^	<.001

*Note*: Mean (± SE) in the same row followed by similar letter (s) is not significantly different (Tukey's HSD test/^†^Games–Howell test: *p* < .05).

### 3.6 Flavonoid content in *R. differens* obtained from different locations

**FIGURE 1:**
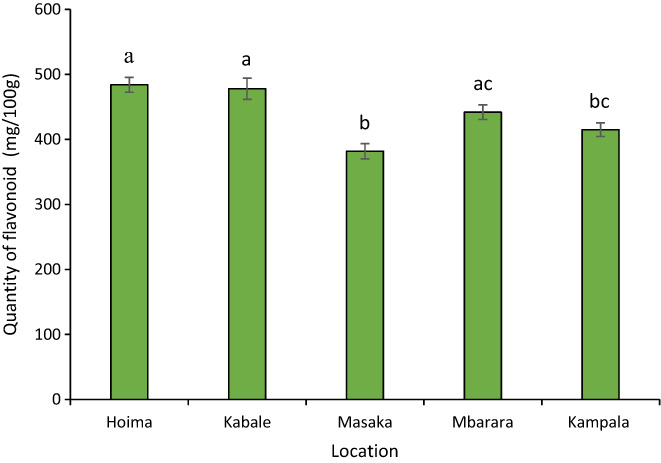
Mean (± SE) of flavonoid composition of *Ruspolia differens* collected from different geographical locations. Bars capped with different letters differed significantly (Tukey's HSD test: *p* < .05).

The flavonoid content of *R. differens* varied by geographical location of collection (*F*_4,10_ = 11.84, *p* < .001). Grasshoppers collected from Hoima (484 ± 11.4 mg/100 g) contained the highest amount of flavonoids while those collected from Masaka (382 ± 11.8 mg/100 g) contained the least quantity (Figure 1).

## 4 DISCUSSION

The findings of this study demonstrate that *R. differens* is rich in macromolecules such as proteins, fats, and carbohydrates. The grasshopper is abundant in essential amino acids, unsaturated fatty acids, minerals, vitamins, and flavonoids that are critical for human health and nutrition. However, the nutrient profile of *R. differens* differed by geographical area of collection. The differences in nutritional profile of insects arise from factors such as developmental stage, origin, and diet of insects (Govorushko, 2019; Kouřimská & Adámková, 2016; Salama, 2020). The swarm of *R. differens* is composed of adult insects which possess wings and therefore able to fly. This implies that the observed variations in nutrient profile did not arise due to differences in the development stage of *R. differens* since swarms comprised adult insects (Matojo & Yarro, 2013). *Ruspolia differens* analyzed in the study were purchased from commercial harvesters from five different districts in Uganda. These districts are found in different agro‐ecological zones which have diverse climatic conditions (Kabasiita et al., 2022). These districts were different from that reported by Ssepuuya et al. (2019), except for Masaka and Kampala. Given that *R. differens* is known to be a highly oligophagous species feeding on diverse host plants that are dominant in their local habitat (Opoke, Malinga, et al., 2019; Valtonen et al., 2018), it is possible that diets might have significantly influenced their nutrient quality (Lehtovaara et al., 2017; Rutaro, Malinga, Lehtovaara, Opoke, et al., 2018; Rutaro, Malinga, Lehtovaara, Valtonen, et al., 2018; Rutaro, Malinga, Opoke, Lehtovaara, et al., 2018). This is further supported by Opoke, Nyeko, et al. (2019) who demonstrated that *R. differens* swarms are usually recruited from locally nonswarming populations known to feed on locally available host plants.

The crude protein content of *R. differens* ranged between 28.2% and 44.8%. This is comparable and higher than protein content of commonly consumed plant and animal proteins such as soybeans, eggs, fish, chicken, beef, and mutton which have a protein content ranging between 13% and 32% (Stadlmayr et al., 2012). The high crude protein content of *R. differens* makes it a suitable alternative that can be used to supplement existing protein sources in the efforts to curb food insecurity and malnutrition especially in Sub‐Saharan Africa (Kekeunou et al., 2020). Crude protein values of *R. differens* obtained from Kabale, Mbarara, and Hoima corroborated findings of other authors (Bbosa et al., 2019; Kinyuru et al., 2010; Siulapwa et al., 2012). However, the crude protein content was lower than values observed by Fombong et al. (2017). The crude fat content of *R. differens* was higher than values recorded for common meat sources (Orkusz, 2021; Williams et al., 2016). Crude fat content of *R. differens* from Masaka was significantly higher than those collected from the other sites. The high crude fat content of *R. differens* obtained from Masaka exceeded previously recorded values for *R. differens* (≤48%) and other edible grasshopper species (Kinyuru et al., 2010; Rumpold & Schlüter, 2013a; Ssepuuya et al., 2017). Crude fat content of *R. differens* from the other sites, however, corroborated findings by some authors (Bbosa et al., 2019; Ssepuuya et al., 2017) and lower than values reported by others (Kinyuru et al., 2010; Siulapwa et al., 2012).

The high carbohydrate content of *R. differens* obtained from the different localities exceeded the previously reported values (1.4%–3.7%) (Bbosa et al., 2019; Fombong et al., 2017; Ssepuuya et al., 2017). These values were, however, within the same range as 7.5%–28.2% recorded in other edible grasshopper species including *Oxya fuscovittata* (Orthoptera: Acrididae), *Oxya hyla* (Orthoptera: Acrididae), and *Boopedon flaviventris* (Orthoptera: Acrididae) (Anand et al., 2008; Blásquez et al., 2012; Ghosh et al., 2016). Carbohydrate content of *R. differens* obtained from Kampala was higher than those collected from the other locations. The high fat and carbohydrate content of the grasshopper supplies energy that is vital for the human body (Hlongwane et al., 2020). Carbohydrates derived from insects are also rich in polysaccharides that enhance immunity in humans (Kouřimská & Adámková, 2016). Limited data were obtained on carbohydrate content of common animal protein sources.

The crude fiber content of *R. differens* from the five districts was lower than quantities (6%–13%) recorded by several researchers (Bbosa et al., 2019; Ssepuuya et al., 2017); but within the same range as the values (4%–5%) observed by Kinyuru et al. (2010). Crude fiber content of *R. differens* from Mbarara was significantly higher than that of grasshoppers obtained from Masaka and Kampala but comparable to those collected from Hoima and Kabale.

*Ruspolia differens* contained more energy compared to beef, chicken, and pork (Orkusz, 2021). Limited data exist on energy content of *R. differens*; however, the values obtained in *R. differens* from the different sites were higher than findings by other researchers (Bbosa et al., 2019). However, the energy content observed in *R. differens* from Hoima, Kabale, Mbarara, and Kampala was lower than values recorded by some authors (Siulapwa et al., 2012). The highest energy content occurred in *R. differens* from Masaka which was probably due to the high crude fats and carbohydrate content of *R. differens* collected from the location.

The lowest ash content observed in *R. differens* collected from Masaka and Kampala was lower than previously recorded values for the species and other edible grasshopper species (Kinyuru et al., 2010; Rumpold & Schlüter, 2013a). The ash content of *R. differens* obtained from Hoima, Kabale, and Mbarara corroborated findings by Kinyuru et al. (2010) but were contrary to findings by other authors who recorded higher (Fombong et al., 2017; Ssepuuya et al., 2017) and lower ash content in *R. differens* (Siulapwa et al., 2012). The ash content of *R. differens* exceeded values recorded in beef, chicken, and pork (Orkusz, 2021; Williams et al., 2016).

The moisture content of *R. differens* collected from the different sites was lower than 50.4%–71.2% as previously reported (Kinyuru et al., 2010; Ssepuuya et al., 2017). The authors analyzed raw, wild‐collected grasshoppers that may have had a higher moisture content compared to the oven‐dried samples analyzed in this study. However, the moisture content of *R. differens* collected from Masaka and Kampala was higher than the previous value obtained for oven‐dried samples (Fombong et al., 2017).

Fatty acids play an integral role in diverse physiological activities in living organisms and supply energy for various functions (De Carvalho & Caramujo, 2018)*. R. differens* recorded high concentration of saturated fatty acids such as palmitic acid, lauric acid, methyl 16 pentacosenoate, methyl heneicosanoate, and methyl nonadecanoate that varied between the areas of collection. Palmitic acid has been observed as the most abundant unsaturated fatty acid in *R. differens*; however, this was only true for *R. differens* obtained from Masaka and Kampala (Malinga et al., 2020; Rutaro, Malinga, Lehtovaara, Opoke, et al., 2018; Rutaro, Malinga, Lehtovaara, Valtonen, et al., 2018). The high quantity of lauric acid, methyl 16 pentacosenoate, methyl heneicosanoate, and methyl nonadecanoate observed in *R. differens* obtained from some of the sites was contrary to the low proportions reported by other researchers (Fombong et al., 2017; Rutaro, Malinga, Lehtovaara, Valtonen, et al., 2018; Ssepuuya et al., 2020). Although oleic acid has been reported as the most abundant monounsaturated fatty acid in *R. differens*, our findings showed that it was only abundant in *R. differens* collected from Mbarara (Cheseto et al., 2020; Rutaro, Malinga, Lehtovaara, Valtonen, et al., 2018; Ssepuuya et al., 2019). Other abundant monounsaturated fatty acids observed in *R. differens* collected from the other areas included elaidolinolenic, methyl cis‐13‐eicosenoate, and methyl cis‐10‐heptadecenoate. These compounds have previously been reported in *R. differens* oil and products made from their oils (Cheseto et al., 2020). Linoleic acid was the most abundant polyunsaturated fatty acid similar to findings by other authors (Cheseto et al., 2020; Malinga et al., 2020; Ssepuuya et al., 2019). Polyunsaturated fatty acids are implicated in the prevention of cancer, cardiovascular diseases, and diabetic neuropathy; however, these compounds cannot be manufactured by mammals and must, therefore, be supplied through diet (Govorushko, 2019; Yorek, 2018). Therefore, the inclusion of *R. differens* can be essential in supplying these polyunsaturated fatty acids.

*Ruspolia differens* samples contained almost all the essential amino acids most of which are not found in plant protein sources (Zielińska et al., 2015). This makes them a suitable alternative to plant proteins for fortification of food products such as flour and baked products in efforts to reduce malnutrition. It can also be utilized as a raw product in food processing to supply essential amino acids (Köhler et al., 2019; Ochieng et al., 2022). Other than leucine, the value of amino acids was lower than quantities obtained from pork, chicken, and beef; and was lower than the daily requirements for adults (Orkusz, 2021; Rumpold & Schlüter, 2013a; WHO, 2007). The inclusion of *R. differens* in diets can, therefore, supplement the essential amino acids supplied by the animal protein sources. The quantities of essential amino acids such as leucine, arginine, threonine, valine, histidine, isoleucine, and phenylalanine did not vary significantly among the five districts. Quantities of leucine obtained were comparable to values reported in Kenya and Uganda but higher than values recorded in *R. differens* collected from Zambia (Fombong et al., 2017; Siulapwa et al., 2012; Ssepuuya et al., 2019). However, the quantities of arginine, threonine, valine, histidine, isoleucine and phenylalanine, methionine, and lysine were lower than previously recorded values (Fombong et al., 2017; Siulapwa et al., 2012; Ssepuuya et al., 2019). Similarly, the quantities of nonessential amino acids such as glutamic acid, aspartic acid, alanine, cysteine, arginine, glycine, proline, serine, and tyrosine detected in *R. differens* collected from the different sites were lower than the values reported by other researchers (Fombong et al., 2017; Siulapwa et al., 2012; Ssepuuya et al., 2019). The low amino acid content recorded in the study could be attributed to the heat processing (oven drying) method that was applied prior to analysis of the samples. Processing is associated with alteration of nutritional profile of *R. differens* (Fombong et al., 2017; Nyangena et al., 2020).

*Ruspolia differens* showed a rich profile of macro‐ and microminerals that are vital for human health and development (Kinyuru et al., 2010; Mwangi et al., 2018; Silva et al., 2019). Minerals function as cofactor for diverse enzymes that are essential for different physiological processes in the human body (Gupta & Gupta, 2014). Minerals are required in diverse quantities and obtained through diet (Silva et al., 2019). Consumption of *R. differens* can, therefore, mitigate mineral deficiencies in humans. Phosphorous content of *R. differens* collected from Hoima, Kabale, Masaka, and Mbarara was lower than the least previously recorded values (121 mg/100) (Kinyuru et al., 2010). The significantly higher value of phosphorus recorded in *R. differens* collected from Kampala was higher than the values observed by Kinyuru et al. (2010) but lower than the findings by Fombong et al. (2017) and Siulapwa et al. (2012). The quantity of phosphorus obtained in *R. differens* collected from Masaka was almost 2.5 times lower than the least recorded quantity in the other sites. Significantly higher potassium content was obtained in *R. differens* collected from Kabale which exceeded the highest value previously observed in the grasshopper (Fombong et al., 2017). The least potassium content occurred in *R. differens* collected from Masaka which was lower than previously recorded value (Kinyuru et al., 2010). The potassium content of *R. differens* obtained from Kampala, Hoima, and Mbarara was comparable to findings documented by other researchers (Fombong et al., 2017; Ssepuuya et al., 2019). A higher content of calcium occurred in grasshoppers from Mbarara followed by Hoima, Kabale, and Kampala. This was comparable to quantities observed by Ssepuuya et al. (2019), higher than the values obtained by Kinyuru et al. (2010), but lower than the findings of other authors (Fombong et al., 2017; Ssepuuya et al., 2017).

The highest zinc content was observed in *R. differens* obtained from Kampala followed by Hoima, Mbarara, and Kabale which concurred with previous reports (Fombong et al., 2017; Kinyuru et al., 2010; Ssepuuya et al., 2019). The significantly lower values of zinc reported in grasshoppers from Masaka was lower than previously observed values for the species; however, the quantity was higher compared to values recorded for *Zonocerus variegatus* (Orthoptera: Pyrgomorphidae), *Melanoplus foedus* (Orthoptera: Acrididae), and *Trimerotropis pallidipennis* (Orthoptera: Acrididae) (Finke, 2015; Ladeji et al., 2003; Oibiokpa et al., 2017). The iron content of *R. differens* analyzed in the study was lower than quantities observed by Fombong et al. (2017). Grasshoppers obtained from Hoima contained the highest iron content followed by those collected from Kabale. These grasshoppers recorded a higher iron content compared to values recorded by Kinyuru et al. (2010) and Ssepuuya et al. (2019) while grasshoppers collected from Mbarara and Kampala contained iron within the same range reported by the authors (13–42 mg/100 g). Grasshoppers collected from Masaka contained significantly lower quantity of iron compared to the other sites albeit higher than the values observed by Siulapwa et al. (2012). The low iron content of *R. differens* from Masaka exceeded the 0.2–0.7 mg/100 g obtained from *Cyrtacanthacris aeruginosa* (Orthoptera: Acrididae), *M. foedus*, and *Z. variegatus* (Alamu et al., 2013; Ladeji et al., 2003; Oibiokpa et al., 2017)*. R. differens* was more abundant in zinc and iron compared to other animal proteins; therefore, the consumption of *R. differens* can curb zinc and iron deficiencies that are prevalent among children and women of childbearing age (Mwangi et al., 2018; Orkusz, 2021).

*Ruspolia differens* collected from the various locations had a rich and varied profile of vitamins such as niacin, riboflavin, folic acid, and pantothenic which occur in abundant quantities in edible insects compared to animal protein sources (Ordoñez‐Araque & Egas‐Montenegro, 2021; Orkusz, 2021). They also contained pyridoxine, retinol, tocopherol, and Vit B12. Vitamins are essential for metabolism in the human body; however, they cannot be manufactured in the body. They are, therefore, continually supplied to human bodies through diet (Alamu et al., 2013; Kekeunou et al., 2020). Inclusion of *R. differens* in diet would, therefore, supplement the food sources of the micronutrients. The highest amount of niacin was obtained from *R. differens* collected from Kabale; however, it was not detected in grasshoppers obtained from Masaka and Kampala. The values observed in the study were higher than previous quantities reported for *R. differens* (Kinyuru et al., 2009) and other edible grasshopper species such as *Sphenarium sp* (Orthoptera: Pyrgomorphidae), *T. pallidipennis*, and *Brachystola magna* (Orthoptera: Romaleidae) (Blásquez et al., 2012; Finke, 2015; Zamudio‐Flores et al., 2019). However, the niacin content of the grasshoppers was lower than the niacin content of *O. hyla hyla* (Ghosh et al., 2016). Riboflavin quantity of *R. differens* collected from Kabale, Masaka, and Kampala was within the same range observed in *R. differens* and other edible grasshoppers (Blásquez et al., 2012; Kinyuru et al., 2009; Oibiokpa et al., 2017). However, *R. differens* collected from Hoima and Mbarara contained higher riboflavin than the previously recorded values.

Higher quantities of pantothenic acid were observed in *R. differens* collected from Kampala, Hoima, and Kabale compared to those obtained from Mbarara and Masaka. The high pantothenic acid content observed in this study corroborates reports that insects are a rich source of pantothenic acid; however, there were limited data on the quantity of pantothenic acid in edible insects (Kumar et al., 2022; Rumpold & Schlüter, 2013a). The quantities of pantothenic acid observed were higher than the values obtained in pallid‐winged grasshoppers and *Acheta domestica* (Orthoptera: Gryllidae) (Finke, 2015; Rumpold & Schlüter, 2013b). The highest pyridoxine content occurred in grasshoppers obtained from Kabale followed by Hoima while those from Mbarara and Kampala recorded comparatively lower values. Higher quantities of pyridoxine were observed in *R. differens* analyzed in the study compared to previous ones (Kinyuru et al., 2009). The pyridoxine quantity in the grasshoppers was equally higher than content observed in other edible grasshoppers (Finke, 2015; Hyun et al., 2012; Zamudio‐Flores et al., 2019). Grasshoppers collected from Kabale, Hoima, and Mbarara recorded higher content of folic acid compared to those obtained from Masaka and Kampala. Folic content of 79–137.6 mg/100 g observed in the study was more than 100 folds higher than quantities recorded for *R. differens* and other grasshopper species (Finke, 2015; Kinyuru et al., 2009; Zamudio‐Flores et al., 2019).

Vitamin B12 content of *R. differens* analyzed exceeded quantities observed in *R. differens* by other authors (Kinyuru et al., 2009; Ssepuuya et al., 2019). It was, however, within the same range as quantities recorded in *M. foedus* (Oibiokpa et al., 2017). Retinol content was higher in *R. differens* collected from Mbarara, Hoima, and Masaka. These quantities were higher than values recorded for *R. differens*, *O. hyla hyla*, and *B. magna* but lower than quantities observed in *M. foedus* (Ghosh et al., 2016; Kinyuru et al., 2009; Oibiokpa et al., 2017). Gamma tocopherol was observed in grasshoppers collected from Hoima, Kabale, and Kampala but was not detected in grasshoppers obtained from Masaka and Mbarara.

Flavonoid content observed in the study was within the same range previously recorded value in processed *R. differens* (Ochieng et al., 2022). Flavonoids are rich sources of antioxidants that possess antimicrobial, anti‐inflammatory, antiallergenic, and anticancer properties, critical for human health (Cheseto et al., 2020; Pal & Dubey, 2013). The flavonoid content of the grasshopper exceeds the quantities of flavonoids obtained in several vegetables and fruits in some countries (Ssepuuya et al., 2020).

Overall, our described patterns of nutritional profile of *R. differens* associations with geographical zones extend our understanding of the ways in which habitat suitability might increasingly affect biodiversity and ecosystem function. It also clearly demonstrates that the nutritional status of the populations of *R. differens* is location specific with limited chances of migrant populations mixing with local populations (Opoke, Malinga, et al., 2019; Valtonen et al., 2018). It motivates further research to evaluate the interaction between locally available host plant types in different *R. differens* swarming areas and to parse the underlying mechanisms and changes in the nutrient status of the various populations. Further, although positive phototaxis in *R. differens* in Uganda has not previously been documented in the literature or studied on artificial lights at night, we found that nocturnal swarming increases *R. differens* density in highly lit areas, thus prompting *R. differens* to alter their flight behavior and navigate toward highly lit urbanized areas that do not contain sufficient quality breeding habitat and food plants on the ground. And when the artificial light cues are absent during the day, the grasshoppers gradually redistribute themselves to areas of vegetative productivity across the landscape. Our observation is strongly supported by several studies, which have clearly demonstrated that grasshoppers drawn to bright environments at night may be ‘trapped’ in the lit area and thereby unable to forage, mate, or disperse into suitable habitat (Degen et al., 2016; Firebaugh & Haynes, 2016; Manfrin et al., 2017; van Langevelde et al., 2017). Although many observations of unidirectional, undistracted migratory flight by grasshoppers have been reported in the literature, none of these cases have been undertaken for *R. differens* tracks and orientation has been estimated over appreciable distance. This observation illustrates the need for further research on artificial light at night effects on the behavior and fitness of *R. differens*. Moreover, this foundational knowledge will illuminate new large‐scale drivers of *R. differens* spatial ecology, revealing insights into the macroscale flows of *R. differens*' biomass and nutrients across the shared landscape in Uganda and other countries.

## 5 CONCLUSION

The nutritional profile of *R. differens* analyzed in the study varied by geographical area of collection. The grasshoppers showed a rich profile of macromolecules, amino acids, saturated and unsaturated fatty acids, minerals, vitamins, and flavonoids; some of which exceeded the values recorded for animal protein sources and recommended daily intake by human adults. The quantities varied from one geographical location to the other. *R. differens* obtained from Kabale had the highest crude protein content while those collected from Masaka had the highest crude fat and carbohydrate content. A total of 37 fatty acids were detected in *R. differens* samples. Linoleic acid was the most abundant PUFA in *R. differens* obtained from Kabale, Masaka, and Mbarara. All essential amino acids were detected in *R. differens* samples. Higher quantities of lysine occurred in *R. differens* from Kabale and Kampala while higher methionine occurred in grasshoppers obtained from Masaka. Higher quantities of macrominerals such as phosphorus, potassium, and calcium were detected in *R. differens* obtained from Kampala, Hoima, and Mbarara, respectively. *R. differens* from Kampala and Hoima recorded higher quantities of zinc and iron, respectively. *R. differens* collected from Kabale recorded the highest quantities of niacin, pyridoxine, and folic acid while grasshoppers obtained from Kampala had the highest quantity of pantothenic acid. The highest flavonoid content was obtained in *R. differens* collected from Hoima. The consumption of *R. differens* can supplement the existing animal protein sources to supply macro‐ and micromolecules that are critical in curbing the rising food security and malnutrition especially in sub‐Saharan Africa.

## AUTHOR CONTRIBUTIONS

**James P. Egonyu:** Project administration (equal); resources (equal); writing – original draft (equal); writing – review and editing (equal). **Margaret Kababu:** Conceptualization (equal); data curation (equal); formal analysis (equal); investigation (equal); methodology (equal); visualization (equal); writing – original draft (equal); writing – review and editing (equal). **Collins K. Mweresa:** Conceptualization (equal); methodology (equal); supervision (equal); writing – original draft (equal); writing – review and editing (equal). **Sevgan Subramanian:** Conceptualization (equal); investigation (equal); writing – review and editing (equal). **Chrysantus M. Tanga:** Conceptualization (equal); funding acquisition (equal); investigation (equal); methodology (equal); project administration (equal); resources (equal); supervision (equal); visualization (equal); writing – original draft (equal); writing – review and editing (equal).

## FUNDING INFORMATION

Financial support for this research was provided by the BioInnovate Africa Programme (INSBIZ—Contribution ID No. 51050076), the Curt Bergfors Foundation Food Planet Prize Award, Bill & Melinda Gates Foundation (INV‐032416), Australian Centre for International Agricultural Research (ACIAR) (ProteinAfrica – Grant No: LS/2020/154), the Curt Bergfors Foundation Food Planet Prize Award, Bill & Melinda Gates Foundation (INV‐032416); the Swedish International Development Cooperation Agency (Sida); the Swiss Agency for Development and Cooperation (SDC); Australian Centre for International Agricultural Research (ACIAR), the Federal Democratic Republic of Ethiopia, and the Government of the Republic of Kenya. Funding was also received from INSEFOODS project, Africa Center for Excellence in Insects Research as food and feeds, Jaramogi Oginga Odinga University of Science and Technology (JOOUST) funded by the World Bank. The funders had no role in study design, data collection and analysis, decision to publish, or preparation of the manuscript. Therefore, the views expressed herein do not necessarily reflect the official opinion of the donors.

## CONFLICT OF INTEREST STATEMENT

The authors declare that they have no conflict of interest.

## Data Availability

All relevant data are within the paper and are available upon request from the authors.
